# Ultrasonographic evaluation of quadriceps femoris muscle mass in critically ill patients aged 60 years and older: a prospective observational study

**DOI:** 10.3389/fragi.2026.1792585

**Published:** 2026-09-24

**Authors:** Nestor-Julian Zarate-Herran, Johan-Sebastián Villada-Gómez, Nestor-David Caicedo, Diana-María Trejos-Gallego, Fernando Gomez

**Affiliations:** 1 4th Year Resident in Internal Medicine and Geriatric Medicine Section, Faculty of Health Sciences, Universidad de Caldas, Manizales, Colombia; 2 Research Group on Geriatrics and Gerontology, Faculty of Health Sciences, Universidad def Caldas, Manizales, Colombia; 3 Intensive Care Unit Hospital Departamental Santa Sofía, Manizales, Colombia; 4 University of Manizales, Manizales, Colombia

**Keywords:** aged, critical illness, intensive care units, muscle wasting, sarcopenia, ultrasonography

## Abstract

**Background and aims:**

Muscle wasting is a common complication in critically ill older adults admitted to intensive care units (ICU), with significant implications for morbidity and mortality. Ultrasound represents a promising bedside tool for assessing muscle mass changes. This study aimed to evaluate changes in quadriceps femoris muscle mass using ultrasonography at ICU admission and after 7 days of stay in patients aged 60 years and older.

**Methods:**

A prospective cohort study with repeated measurements was conducted between December 2024 and May 2025. Patients aged ≥60 years admitted to the ICU were included. Ultrasonographic measurements included rectus femoris cross-sectional area (RFCSA), bilateral quadriceps muscle thickness, and rectus femoris pennation angle at admission and day 7. Clinical characteristics, severity scores (APACHE II, SOFA), and functional status (Barthel index) were recorded. Statistical analysis included Wilcoxon signed-rank test and robust statistics.

**Results:**

Fifty patients were analyzed (60% male, median age 72.5 years). *Significant* muscle mass reductions were observed: RFCSA decreased by 16.2% (2.525–2.115 cm^2^, p < 0.001), right quadriceps thickness by 14.6% (2.012–1.719 cm, p < 0.001), left quadriceps thickness by 20.2% (2.049–1.635 cm, p < 0.001), and pennation angle by 18.2% (7.664°–6.270°, p < 0.001). ICU mortality was 30%. Multivariate analysis identified advanced age, female sex, hypoglycemic agent use, higher SOFA scores, and lower premorbid Barthel index as factors associated with greater muscle loss.

**Conclusion:**

Critically ill older adults experience significant quadriceps muscle mass loss within the first week of ICU stay, as measured by bedside ultrasonography. These findings support the implementation of early muscle assessment and targeted interventions in this vulnerable population.

## Introduction

The increasing life expectancy worldwide has resulted in a rapidly growing population of adults aged 60 years and older, with projections indicating that one in six people globally will be over 60 years by 2030 (World Health Organization, 2024). This demographic shift has led to increased utilization of high-complexity hospital services, necessitating enhanced research to expand knowledge of health conditions affecting individuals in this life stage (Ribera Casado, 2001).

Approximately 25% of adults over 65 years are hospitalized annually, with significant impacts on the development of various geriatric syndromes including polypharmacy, sarcopenia, delirium, malnutrition, and frailty (Gómez Montes et al., 2021). In the United States, 48% of patients requiring intensive care unit (ICU) admission are over 65 years old, constituting a population with special needs that directly influence their morbidity and mortality (Carson, 2003). Among older adults requiring ICU stay, muscle wasting represents a medical problem of global impact (Schefold et al., 2020).

Skeletal muscle provides the primary site for amino acid uptake and utilization, facilitates locomotion, and contributes to whole-body metabolism (Mukund and Subramaniam, 2020). Representing approximately 40% of total body mass, muscle tissue reduction increases the likelihood of developing additional health complications, resulting in decreased quality of life (Cruz-Jentoft et al., 2019). Among older adults admitted to ICU, low skeletal muscle mass prevalence reaches 51%, with this condition increasing mortality risk with a combined odds ratio of 2.35 (95% CI, 1.91–2.89) compared to those with normal muscle mass (Yang et al., 2023).

Muscle atrophy and protein catabolism are promoted by various factors frequently encountered in ICU patients, including insulin resistance and hyperglycemia, inflammation, muscle disuse due to immobilization, cytokine action, pro-inflammatory medication use, delayed or difficult nutritional support, and advanced age (Friedrich et al., 2015). The increase in muscle catabolism during ICU stay is crucial, as it is essential for coagulation factor synthesis, acute phase protein production, and serves as an energy substrate for immune system cells and gluconeogenesis (Van Gassel et al., 2020).

During the initial 2 weeks of ICU stay there is a protein loss between 10% and 20% (Gamrin-Gripenberg et al., 2018).

Muscle mass loss during ICU stay begins early and accelerated, with daily reductions of up to 3% (Gamrin-Gripenberg et al., 2018). Ultrasonographic assessment has identified differential atrophy patterns between muscle groups, with rectus femoris thickness and cross-sectional area decreasing daily 1.75% and 2.1%, respectively (Fazzini et al., 2023).

However, precise muscle atrophy measurement in ICU settings represents a significant challenge (Vanhorebeek et al., 2020). While gold standard modalities for muscle mass determination include magnetic resonance imaging, computed tomography, and X-ray absorptiometry, these techniques are impractical in ICU settings due to high costs, radiation exposure, or lack of portability (Ceniccola et al., 2019). Consequently, bedside tools that are sensitive, specific, safe, cost-effective, and user-friendly are required for quantifying muscle atrophy in older adults, positioning ultrasonography as a key tool in this process (Nakanishi et al., 2020).

Despite the clinical relevance of muscle wasting in critically ill older adults, limited scientific evidence exists regarding peripheral muscle ultrasonography use for evaluating muscle mass loss in this vulnerable population during ICU stay (López Jiménez et al., 2024). The aim of this study was to quantify changes in quadriceps femoris cross-sectional area, muscle thickness, and pennation angle between ICU admission and day 7 in older adults, and to evaluate their associations with clinical and functional characteristics and severity scores in an ICU located in the Andes Mountains.

## Materials and methods

### Study design and setting

A prospective analytical cohort study with repeated measurements was conducted at ICU in a tertiary hospital in Manizales, located in the Colombian Andes Mountains region, from December 2024 to May 2025.

The inclusion criteria comprised patients aged 60 years or older admitted to the ICU for medical or surgical conditions. Exclusion criteria included: limitation of life-sustaining treatment or therapeutic redirection orders, burn injuries, a FLUID Protocol score ≥ 2 — assessed at both ICU admission and day 7 of ICU stay to identify and exclude patients with significant subcutaneous edema at either time point—and failure to provide signed informed consent.

The required sample size to detect changes in quadriceps femoris muscle mass in adults aged ≥60 years admitted to the ICU was calculated using a paired mean comparison formula for longitudinal studies with repeated measurements: n = [(Zα/2 + Zβ)^2^ × σ^2^ × (1 − ρ)]/d^2^, where Zα/2 = 1.96 (95% confidence level), Zβ = 1.28 (90% statistical power), σ = expected standard deviation of the change in muscle mass (1.5 cm), ρ = correlation between baseline and day-7 measurements (0.5), and d = minimum clinically relevant difference to be detected (0.5 cm). The standard deviation (σ = 1.5 cm) was estimated based on prior studies reporting variability ranging from 0.8 to 1.5 cm in older ICU patients (Puthucheary et al., 2013; Weinel et al., 2019). The resulting calculated sample size was approximately 48 patients.

All patients underwent ultrasonographic evaluation within 48 h of ICU admission and again at day 7 of ICU stay. Informed consent was obtained from all participants or their legally authorized representatives, and the study was approved by the local institutional ethics committee.

### Ultrasonographic protocol

Ultrasonographic examinations were performed using a high-frequency linear transducer in musculoskeletal mode (Philips Ultrasound, Bothell, WA) with factory harmonic settings. Gain was set between 50% and 70%, and depth was adjusted to visualize the femoral cortex or deep aponeurosis of the evaluated muscle group. B-mode was employed for all measurements (Buitrago et al., 2024).

The rectus femoris was evaluated by measuring the distance between the right anterior superior iliac spine and the superior patellar border using a rigid measuring tape. Two specific points were marked: midpoint (50% of total distance) and distal third (66.6% of total distance) (Formenti et al., 2019).

### Measurements

Rectus femoris cross-sectional area (RFCSA): Measured at the distal third of the right lower limb using a transverse axis transducer with adequate transmission gel and minimal compression. Clear muscle visualization was obtained, the image was frozen, and the area was delimited in cm^2^ using the ultrasound software planimetry function. Three consecutive measurements were performed, and the average was recorded.

Quadriceps muscle thickness: Measured at the midpoint and distal third of both lower limbs using a transverse axis with minimal compression. The frozen image was used to identify the distance between the femoral cortex and the inferior border of the rectus femoris superior fascia. The transducer was applied twice at each point, and the average was calculated.

Rectus femoris pennation angle: Measured at the distal third using longitudinal axis insonation with minimal compression, positioned parallel to the skin. The transducer was then tilted laterally 15°–30°, the image was frozen, and the angle between muscle fascicles and the inferior aponeurosis was calculated using the ultrasound equipment’s angle measurement tool.

All ultrasonographic evaluations were performed by a critical care specialist with more than 5 years of ultrasonography experience.

### Data collection

For this observational study, were collected general patient characteristics, ICU outcomes, risk factors associated with Muscle Wasting previously reported in the literature (Hermans and Van den Berghe, 2015). Variables included demographic characteristics (age, sex, social security affiliation), anthropometric measurements (weight, height, BMI), comorbidities, ICU length of stay, severity scores (APACHE II, SOFA), pre-admission functional status (Barthel index), Charlson comorbidity index, and mortality outcomes.

### Statistical analysis

Descriptive analysis included absolute and relative distributions and summary measures. Normality was assessed using Shapiro-Wilk test and Q-Q plots; homoscedasticity was evaluated using Levene’s test. For muscle mass variation analysis, Wilcoxon signed-rank test was applied with Hodges-Lehmann estimator and rank biserial effect size. Robust statistics included Yuen’s t-test for paired samples with Algina-Keselman-Penfield (AKP) robust effect size estimator. Correlations were assessed using Spearman correlation coefficients and partial correlations. Multivariable analysis was performed using median (0.5) quantile regression, selected for its robustness to heteroscedasticity and outliers. Initially, exploratory univariable analyses assessed the association between each covariate and the outcomes (changes in RFCSA, QMT, and RFPA). Variables demonstrating statistical significance or a clinically relevant trend were considered for multivariable modeling. Additionally, variables with established clinical plausibility as determinants of muscle loss in critically ill patients were included irrespective of univariable significance (age, sex, SOFA score and premorbid Barthel Index). Candidate variables identified through these criteria were subsequently entered into a stepwise selection procedure within the quantile regression framework.

## Results

### Study population

A total of 50 patients were included in the final analysis (Table 1). The median age was 72.5 years (interquartile range [IQR] 70–76 years), with male participants comprising 60% (n = 30) of the study population. The median body mass index was 22.4 kg/m^2^ (IQR 22.1–23.3 kg/m^2^). Regarding healthcare coverage, 18% of patients (n = 9) belonged to the contributory social security regime, while 82% (n = 41) were affiliated with the subsidized regime.

**TABLE 1:** Demographic and clinical characteristics of the studied patients.

Demographic	
Female, n (%)	20 (40%)
Age (years), median (IQR)	72.5 (70–76)
Health insurance regimen (%)	
Subsidized	41 (82%)
Contributory	9 (18%)
Clinical	
Hospital stays (days), median (IQR)	16.5 (13–20)
Mortality in ICU, n (%)	15 (30)
Coexisting diseases, n (%)	
Systemic arterial hypertension	37 (74)
Dyslipidemia	19 (38)
Diabetes mellitus	12 (24)
Hypothyroidism	12 (24)
Aortic valvular heart disease	10 (20)
Ischemic heart disease	6 (12)
Chronic obstructive pulmonary disease	6 (12)
Chronic kidney disease	5 (10)
Cardiac arrhythmia	3 (6)
Body weight (kg), median (IQR)	59
Height (m), median (IQR)	1.62
Body mass index (kg/m^2^), median (IQR)	22.4 (22.1–23.3)
APACHE II, median, IQR	19 (16–21)
SOFA, median, IQR	6 (4–9)
Barthel index at admission, median, IQR	100 (90–100)
Charlson comorbidity index, median, IQR	4 (3–5)
Pharmacological, n (%)	
Antihypertensives	33 (66)
Statins	18 (36)
Hypothyroidism	12 (24)
Antiplatelet/anticoagulants	12 (24)
Hypoglycemic agents/insulins	11 (22)
Gastroprotection	8 (16)
Analgesics	6 (12)
Inhalation therapy	4 (8)
Antidepressants	2 (4)
Hypnotics/sedatives/antipsychotics	2 (4)
Number of medications, median (IQR)	3 (2–4)

The most prevalent comorbidities were systemic arterial hypertension in 37 patients (74%), followed by dyslipidemia in 19 patients (38%), diabetes mellitus and hypothyroidism each affecting 12 patients (24%), and valvular heart disease in 10 patients (20%). Less frequent comorbidities included ischemic heart disease and chronic obstructive pulmonary disease, each present in 6 patients (12%), chronic kidney disease in 5 patients (10%), and cardiac arrhythmia in 3 patients (6%). The median Charlson Comorbidity Index was 4.0 (IQR 3.0–5.0), indicating a moderate to high comorbidity burden in the study population.

### Clinical characteristics and severity scores

At ICU admission, the median APACHE II score was 19.0 (IQR 16.0–21.0), and the median SOFA score was 6.0 (IQR 4.0–9.0). The median Barthel functional index was 100.0 (IQR 90.0–100.0), indicating functional independence to mild dependence prior to ICU admission.

The median ICU length of stay was 16.5 days (IQR 13.0–20.0). ICU mortality occurred in 15 patients (30%).

### Ultrasonographic muscle mass changes

Significant reductions in all ultrasonographic parameters were observed between admission and day 7 of ICU stay (Table 2).

**TABLE 2:** Ultrasonographic parameters: baseline vs. day 7.

Parameter	Baseline median (IQR)	Day 7 median (IQR)	Change (%)	p-value
RFCSA (cm^2^)	2.525 (1.950–3.200)	2.115 (1.630–2.680)	−16.2%	<0.001
Right QT (cm)	2.012 (1.650–2.400)	1.719 (1.400–2.050)	−14.6%	<0.001
Left QT (cm)	2.049 (1.680–2.450)	1.635 (1.320–2.100)	−20.2%	<0.001
Penn Angle (°)	7.664 (6.200–9.100)	6.270 (5.100–7.800)	−18.2%	<0.001

RFCSA: Rectus femoris cross-sectional area; QT: quadriceps thickness; Penn Angle: Pennation angle.

### Robust statistical analysis

Yuen’s robust t-test confirmed these findings with similar effect sizes: RFCSA (AKP effect size = 0.89), right quadriceps thickness (AKP = 0.81), left quadriceps thickness (AKP = 0.93), and pennation angle (AKP = 0.77), all indicating large effect sizes.

### Correlation analysis

Spearman correlation analysis revealed significant associations between muscle mass changes and clinical variables. Greater muscle mass loss was associated with:

- Age: Moderate positive correlation with RFCSA reduction (r = 0.42, p = 0.003)
- Female sex: Significantly greater muscle loss in women (median RFCSA reduction: −18.5% vs. −14.1% in men, p = 0.024)
- SOFA score: Higher severity scores correlated with greater muscle loss (r = 0.38, p = 0.007)
- Barthel index: Lower baseline functional status associated with greater muscle wasting (r = −0.35, p = 0.013)

### Multivariate analysis of factors associated with femoral quadriceps muscle mass changes

The multivariate analysis revealed several significant predictors of femoral quadriceps muscle mass changes across different ultrasound parameters (Table 3).

**TABLE 3:** Multivariate models to identify the factors with the greatest effect on the change in quadriceps femoris muscle mass.

Quadriceps femoris muscle mass	Factors	Coefficient (C.I. 95%)	T-test [p-value]
RFCSA (cm^2^) Pseudo R^2^: 0.3732 Minimum sum deviations: 6.4052 Sum of deviations: 10.2195	Age	−0.0227 (−0.0381 a −0.0072)	−2.95 [0.005]
	Sex (male)	0.3885 (0.0945 a 0.6825)	2.66 [0.011]
	Hypoglycemic agents	−0.3480 (−0.6697 a −0.0263)	−2.18 [0.035]
Right QT (cm) Pseudo R^2^: 0.2785 Minimum sum deviations: 7.4441 Sum of deviations: 10.3181	Sex (male)	0.6978 (0.4438 a 0.9518)	5.53 [<0.001]
	Barthel	0.0151 (0.0043 a 0.0260)	2.81 [0.007]
	SOFA	−0.0433 (−0.0766 a −0.0100)	−2.62 [0.012]
Left QT (cm) Pseudo R^2^: 0.3352 Minimum sum deviations: 6.4074 Sum of deviations: 9.6374	Age	−0.0167 (−0.0313 a −0.0022)	−2.33 [0.025]
	Sex (male)	0.5588 (0.2797 a 0.8379)	4.04 [<0.001]
	# Medications	0.1054 (0.0149 a 0.1958)	2.35 [0.024]
	Hypoglycemic agents	−0.3294 (−0.5795 a −0.0793)	−2.66 [0.011]
	Statins	−0.3374 (−0.6381 a −0.0366)	−2.26 [0.029]
	Antihypertensives	−0.3803 (−0.7956 a 0.0350)	−1.85 [0.072]
Penn angle (°) Pseudo R^2^: 0.2202 Minimum sum deviations: 20.9041 Sum of deviations: 26.8062	Sex (male)	1.1134 (0.6247 a 1.6021)	4.59 [<0.001]
	Mortality in the unit	−1.0913 (−1.4417 a −0.7409)	−6.27 [<0.001]
	High blood pressure	−0.5593 (−1.1076 a −0.0109)	−2.05 [0.046]

RFCSA: Rectus femoris cross-sectional area; QT: quadriceps thickness; Penn Angle: Pennation angle.

Male sex emerged as the most consistent and robust predictor, demonstrating significant positive associations across all muscle measurements: cross-sectional area of the rectus femoris (ASTRF: β = 0.3885, p = 0.011), mid-thigh thickness at the midpoint (GCF_MID: β = 0.6978, p < 0.001), mid-thigh thickness at the distal point (GCF_MII: β = 0.5588, p < 0.001), and pennation angle of the rectus femoris (APRF: β = 1.1134, p < 0.001).

Age demonstrated a significant negative association with muscle mass preservation, showing detrimental effects on both ASTRF (β = −0.0227, p = 0.005) and GCF_MII (β = −0.0167, p = 0.025), indicating that older patients experienced greater muscle mass loss during ICU stay.

Clinical severity scores showed differential impacts depending on the muscle parameter assessed. Higher SOFA scores were associated with greater muscle loss in GCF_MID measurements (β = −0.0433, p = 0.012), while better functional status as measured by the Barthel Index was associated with muscle mass preservation (β = 0.0151, p = 0.007).

Medication-related factors demonstrated significant associations with muscle changes. Hypoglycemic agents showed consistent negative effects on both ASTRF (β = −0.3480, p = 0.035) and GCF_MII (β = −0.3294, p = 0.011), while statin therapy was associated with reduced muscle thickness (GCF_MII: β = −0.3374, p = 0.029). Conversely, polypharmacy, as measured by the total number of medications, showed a modest positive association with muscle thickness (GCF_MII: β = 0.1054, p = 0.024).

ICU mortality emerged as a strong predictor of muscle architecture changes, with deceased patients showing significantly reduced pennation angles (APRF: β = −1.0913, p < 0.001). Additionally, systemic arterial hypertension was associated with decreased pennation angles (β = −0.5593, p = 0.046).

The models demonstrated moderate explanatory power, with pseudo R^2^ values ranging from 0.2202 to 0.3732, indicating that the identified factors account for a substantial portion of the variance in muscle mass changes during critical illness.

## Discussion

This prospective observational study demonstrates that critically ill older adults experience rapid and clinically significant skeletal muscle loss within the first week of ICU admission, as quantified by bedside ultrasonography. Rectus femoris cross-sectional area decreased by 16.2%, quadriceps muscle layer thickness decreased by 14.6%–20.2%, and pennation angle decreased by 18.2%, collectively indicating both quantitative and qualitative muscle deterioration that exceeds accepted thresholds of measurement variability. These findings are consistent with the accelerated catabolism characteristic of critical illness, during which protein losses may reach up to 3% of total body protein stores per day (Gamrin-Gripenberg et al., 2018), and corroborate observations reported in comparable patient populations (Puthucheary et al., 2013; Parry et al., 2015).

The reduction in pennation angle suggests architectural remodeling of skeletal muscle, potentially reflecting alterations in muscle fiber orientation and impaired contractile capacity (Parry et al., 2015; Lee et al., 2021; Umbrello et al., 2024). These results are further supported by prior studies documenting a 17.7% reduction in rectus femoris cross-sectional area (Puthucheary et al., 2013) and reductions in muscle layer thickness of up to 30% among mechanically ventilated patients within 10 days of ICU admission (Parry et al., 2015).

Importantly, our focus on older adults, together with the observed association between advanced age and greater muscle mass loss, underscores their increased vulnerability, driven by baseline sarcopenia, multimorbidity, and impaired protein metabolism (Nijholt et al., 2017). From a pathophysiological perspective, accelerated muscle wasting in this population likely reflects the combined effects of systemic inflammation, insulin resistance, immobilization, suboptimal nutritional intake, and pharmacologic exposures, superimposed on age-related hormonal decline, mitochondrial dysfunction, and pre-existing sarcopenia (Friedrich et al., 2015; Nakanishi et al., 2020; McKendry et al., 2020). Female sex as an independent risk factor is particularly noteworthy, as it may reflect differences in baseline muscle mass, hormonal influences, or differential responses to critical illness stress (Wu et al., 2022; Deutz et al., 2023).

The muscle mass reduction observed in our cohort is consistent with recent ultrasonographic studies in ICU populations. Puthucheary et al. (2013) reported rectus femoris cross-sectional area reductions of 17.7% within 10 days of ICU admission in a mixed-age population. Similarly, Parry et al. (2015) documented 30% thickness reduction in mechanically ventilated patients over 10 days. However, our study specifically focused on older adults, a population particularly vulnerable to muscle wasting due to age-related sarcopenia, comorbidity burden, and altered protein metabolism (Nijholt et al., 2017).

The strong association between muscle loss and mortality further supports ultrasonographic assessment as a potential prognostic biomarker (Formenti et al., 2019). A novel finding of this study was the association between lower pennation angle and mortality. Although recent evidence indicates that greater reductions in pennation angle are associated with accelerated muscle thickness loss and the development of intensive care unit–acquired weakness (ICU-AW) (Fazzini et al., 2023), its association with mortality has not been previously reported. Frailty may represent a plausible pathophysiological link underlying this association (Yurumez et al., 2024), however, because frailty was not formally assessed in our cohort, this hypothesis remains speculative and warrants confirmation in future studies.

These findings have relevant clinical implications. First, the early onset and substantial magnitude of muscle loss during the first week of ICU stay support the need for prompt identification and intervention. The feasibility of bedside ultrasonography for muscle assessment offers a practical and reproducible method for real-time monitoring of muscle mass changes (Parry et al., 2015). Second, the identification of factors associated with greater muscle loss—advanced age, female sex, higher SOFA scores, reduced baseline functional status, and use of hypoglycemic agents—allows risk stratification and the implementation of targeted preventive strategies in high-risk patients (Friedrich et al., 2015; Vanhorebeek et al., 2020). At this stage, it is important to emphasize that not only markers of acute physiological dysfunction, such as the SOFA score, but also pre-admission factors—including baseline functional status—are independently associated with worse outcomes and greater muscle mass loss. These findings reinforce the relevance of pre-existing vulnerability in modulating the impact of critical illness. Third, the observed association between muscle loss and mortality suggests that ultrasonographic muscle assessment may have prognostic value and could contribute to clinical decision-making, including intensity of care, rehabilitation planning, and communication with families (Formenti et al., 2019).

## Methodological strengths

This study presents several methodological strengths. The prospective design with standardized measurement protocols minimizes bias and ensures data quality. The use of robust statistical methods, including Hodges-Lehmann estimators and quantile regression, provides reliable estimates in the presence of non-normal distributions and outliers commonly encountered in ICU populations.

The standardized ultrasonographic protocol with multiple measurements and experienced operator reduces measurement variability. The comprehensive clinical characterization including severity scores, comorbidity indices, and functional assessment enables thorough exploration of factors associated with muscle loss.

## Limitations

Several limitations of this study warrant consideration. First, the single-center design and relatively small sample size may restrict the generalizability of findings to broader or more heterogeneous populations, and may limit statistical power for subgroup analyses and multivariable modeling. Second, muscle function was not assessed, precluding definitive conclusions regarding the functional significance of the observed morphological changes. Third, nutritional intake and physical therapy interventions were not systematically quantified, which may have introduced confounding in the relationship between muscle loss and clinical outcomes. Fourth, the absence of a validated frailty assessment instrument limits the interpretability of frailty as a potential determinant of muscle mass loss and clinical outcomes. This gap arose, in part, from limited familiarity with frailty assessment tools among critical care specialists, as well as the practical constraints inherent to their administration in the ICU setting—underscoring the need for an interdisciplinary approach that incorporates geriatric expertise in both the clinical evaluation and systematic study of critically ill older adults. Finally, the 7-day follow-up period, while appropriate for capturing early muscle changes, may be insufficient to characterize the full trajectory of muscle recovery or to assess longer-term functional and clinical outcomes.

## Future directions

Future research should focus on several key areas. Larger multicenter studies are needed to confirm these findings and develop validated prediction models for muscle wasting risk. Integration of muscle function assessments with morphological measurements would provide a more comprehensive understanding of muscle changes during critical illness.

Investigation of targeted interventions including early mobilization protocols, optimized nutrition strategies, and pharmacological approaches to prevent muscle wasting represents a priority research area. Additionally, exploring the relationship between early muscle changes and long-term functional outcomes would inform rehabilitation planning and resource allocation.

## Conclusion

This prospective observational study demonstrates that critically ill older adults develop rapid and clinically significant quadriceps muscle loss within the first week of ICU admission, as quantified by bedside ultrasonography. Significant reductions were observed in rectus femoris cross-sectional area, bilateral quadriceps muscle layer thickness, and pennation angle; moreover, the magnitude of muscle loss was independently associated with increased mortality. Advanced age, female sex, greater illness severity, lower baseline functional status, and use of hypoglycemic agents were identified as independent factors associated with accelerated muscle wasting—collectively underscoring that both acute physiological derangement and pre-existing vulnerability contribute to muscle loss and adverse clinical outcomes.

Bedside ultrasonographic muscle assessment proved to be a feasible and non-invasive method for real-time monitoring and demonstrated prognostic value in this population. Routine integration of quantitative muscle assessment into ICU practice may facilitate early risk stratification and inform targeted interventions, including optimized nutritional support, early mobilization, and individualized rehabilitation strategies. Future studies are warranted to validate these findings in larger, multicenter cohorts and to determine whether interventions aimed at attenuating muscle loss translate into meaningful improvements in survival and functional recovery.

## Data Availability

The datasets presented in this study can be found in online repositories. The names of the repository/repositories and accession number(s) can be found below: https://repositorio.ucaldas.edu.co/entities/person/cc63a3f8-918d-45bf-8207-91e5a7d043a2.
